# How Can Research on Artificial Empathy Be Enhanced by Applying Deepfakes?

**DOI:** 10.2196/29506

**Published:** 2022-03-04

**Authors:** Hsuan-Chia Yang, Annisa Ristya Rahmanti, Chih-Wei Huang, Yu-Chuan Jack Li

**Affiliations:** 1 Graduate Institute of Biomedical Informatics College of Medical Science and Technology Taipei Medical University Taipei Taiwan; 2 International Center for Health Information Technology Taipei Medical University Taipei Taiwan; 3 Research Center of Big Data and Meta-analysis Wan Fang Hospital Taipei Medical University Taipei Taiwan; 4 Clinical Big Data Research Center Taipei Medical University Hospital Taipei Taiwan; 5 Department of Health Policy Management Faculty of Medicine, Public Health, and Nursing Universitas Gadjah Mada Yogyakarta Indonesia; 6 Department of Dermatology Wanfang Hospital Taipei Taiwan

**Keywords:** artificial empathy, deepfakes, doctor-patient relationship, face emotion recognition, artificial intelligence, facial recognition, facial emotion recognition, medical images, patient, physician, therapy

## Abstract

We propose the idea of using an open data set of doctor-patient interactions to develop artificial empathy based on facial emotion recognition. Facial emotion recognition allows a doctor to analyze patients' emotions, so that they can reach out to their patients through empathic care. However, face recognition data sets are often difficult to acquire; many researchers struggle with small samples of face recognition data sets. Further, sharing medical images or videos has not been possible, as this approach may violate patient privacy. The use of deepfake technology is a promising approach to deidentifying video recordings of patients’ clinical encounters. Such technology can revolutionize the implementation of facial emotion recognition by replacing a patient's face in an image or video with an unrecognizable face—one with a facial expression that is similar to that of the original. This technology will further enhance the potential use of artificial empathy in helping doctors provide empathic care to achieve good doctor-patient therapeutic relationships, and this may result in better patient satisfaction and adherence to treatment.

## Introduction

Good doctor-patient communication is one of the key requirements of building a successful, therapeutic doctor-patient relationship [1]. This type of communication enables physicians to provide better-quality care that may impact patients’ health. Studies on good doctor-patient communication have demonstrated a strong positive correlation between physician communication skills and patient satisfaction, which is likely associated with patients’ adherence to treatment; their experience of care; and, consequently, improved clinical outcomes [2-5].

We acknowledge the importance of good doctor-patient communication; doctors must understand patients’ perspectives through verbal conversation and nonverbal behaviors (eg, posture, gesture, eye contact, facial expression, etc) [6,7]. Establishing communication involving nonverbal messages is very important in building a good doctor-patient relationship because such communication conveys more expressive and meaningful messages than those conveyed in a verbal conversation [8]. One research study indicates that nonverbal messages contribute to up to 90% of messages delivered in human interactions [6]. Another study also estimates that more than half of outpatient clinic patients believe that establishing positive nonverbal behaviors indicates that a doctor is more attentive toward their patient and thus results in better patient satisfaction and adherence to treatment [8].

Although several studies have reported that human nonverbal behaviors are significantly associated with patient satisfaction and compliance to a treatment plan, physicians are often clueless about nonverbal messages [6]. Doctors should be more aware of their nonverbal behaviors because patients are cognizant of them. Doctors also need to recognize and evaluate patients’ nonverbal behaviors and their own nonverbal behaviors toward patients.

Artificial intelligence (AI) offers great potential for exploring nonverbal communication in doctor-patient encounters [9]. For example, AI may help a doctor become more empathic by analyzing human facial expressions through emotion recognition. Once an emotionally intelligent AI identifies an emotion, it can guide a doctor to express more empathy based on each patient’s unique emotional needs [10].

Empathy refers to the ability to understand or feel what another person is experiencing, and showing empathy may lead to better behavioral outcomes [9]. Empathy can be learned, and the use of AI technology introduces a promising approach to incorporating artificial empathy in the doctor-patient therapeutic relationship [11]. However, human emotions are very complex. An emotionally intelligent AI should learn a range of emotions (ie, those that patients experience) from facial expressions, voices, and physiological signals to empathize with human emotions [12]. These emotions can be captured by using various modalities, such as video, audio, text, and physiological signals [13].

Among all forms of human communication channels, facial expressions are recognized as the most essential and influential [14-16]. The human face can express various thoughts, emotions, and behaviors [15]. It can convey important aspects in human interpersonal communication and nonverbal expressions in social interactions [17,18]. Compared to the amount of information that can be conveyed via emotion recognition technology, facial expressions convey 55% of the emotional expression transmitted in multimodal human interactions, whereas verbal information, text communication, and communication via physiological signals only convey 20%, 15%, and 10% of the total information in interactions, respectively [19].

Many researchers have been studying facial expressions by using automatic facial emotion recognition (FER) to gain a better understanding of the human emotions linked with empathy [20-24]. They have proposed various machine learning algorithms, such as support vector machines, Bayesian belief networks, and neural network models, for recognizing and describing emotions based on observed facial expressions recorded on images or videos [20-22]. Although mounting literature has been introduced on machine learning and deep learning for automatically extracting emotions from the human face, developing a highly accurate FER system requires a lot of training data and a high-quality computational system [21]. In addition, the data set must include diverse facial views in terms of angles, frame rates, races, and genders, among others [21].

Many public data sets are available for FER [25]. However, most public data sets are not sufficient for supporting doctor-patient interactions. Creating our own medical data sets is also not possible, since this process is expensive and time consuming [26]. Moreover, researchers often struggle with acquiring sufficient data for training a face recognition model due to privacy concerns. Data sharing and the pooling of medical images or videos are not even possible, as these approaches may violate patient privacy. Herein, we present our study on the emerging AI is known as *deepfakes*—a technology that enables face deidentification for recorded videos of patients’ clinical encounters. This technology can revolutionize FER by replacing patients’ faces in images or videos with an unrecognizable face, thereby anonymizing patients. This could protect patients’ privacy when it comes to clinical encounter videos and allow medical video data sharing to become more feasible. Moreover, using an open clinical encounter video data set can also promote more in-depth research within the academic community. Thus, deepfake technology will further enhance the clinical application of artificial empathy for medical application purposes.

## Methods

### Human FER

Human FER plays a significant role in understanding people's nonverbal ways of communicating with others [19]. It has attracted the interest of scientific populations in various fields due to its superiority among other forms of emotion recognition [22]. As it is not only limited to human-computer interactions or human-robot interactions, facial expression analysis has become a popular research topic in various health care areas, such as the diagnosis or assessment of cognitive impairment (eg, autism spectrum disorders in children), depression monitoring, pain monitoring in Parkinson Disease, and clinical communication in doctor-patient consultations [27].

The main objective of FER is to accurately classify various facial expressions according to a person’s emotional state [21]. The classical FER approach is usually divided into the following three major stages: (1) facial feature detection, (2) feature extraction, and (3) emotion recognition [21,28]. However, traditional FER has been reported to be unable to extract facial expressions in an uncontrolled environment with diverse facial views [21,28]. On the other hand, a recent study using a deep learning–based FER approach has successfully achieved superior accuracy over that of traditional FER [20-22].

### Deepfake Technology

The rapid growth of computer vision and deep learning technology has driven the recently emerged phenomena of deepfakes (*deep learning* and *fake*), which can automatically forge images and videos that humans cannot easily recognize [29-31]. In addition, deepfake techniques offer the possibility of generating unrecognizable images of a person’s face and altering or swapping a person’s face in existing images and videos with another face that exhibits the same expressions as the original face [29]. Various deepfake attempts have been used for negative purposes, such as creating controversial content related to celebrities, politicians, companies, and even individuals to damage their reputation [30]. Although the harmful effects of deepfake technology have raised public concerns, there are also advantages to using this technology. For example, it can provide privacy protection in some critical medical applications, such as face deidentification for patients [32]. Further, although deepfake technology can easily manipulate the low-level semantics of visual and audio features, a recent study suggested that it might be difficult for deepfake technology to forge the high-level semantic features of human emotions [31].

Deepfake technology is mainly developed by using deep learning—an AI-based method that can be used to train deep networks [29]. The popular approach to implementing deepfake techniques is based on the generative adversarial network (GAN) model [33,34]. There are several types and examples of deepfakes, such as photo deepfakes, audio deepfakes, video deepfakes, and audio-video deepfakes.

### Data Set

To simulate how deepfake technology enables face deidentification for recorded videos of doctor-patient clinical encounters, we recruited 348 adult patients and 4 doctors from Taipei Municipal Wanfang Hospital and Taipei Medical University Hospital from March to December 2019. After excluding video data from 21 patients due to video damage, we collected video data from 327 patients. The data set focused on the interactions between doctors and patients in dermatology outpatient clinics. The subjects in the data set are all from the Taiwanese population.

### The FER System in the Deepfake Model Setup

**Figure 1:**
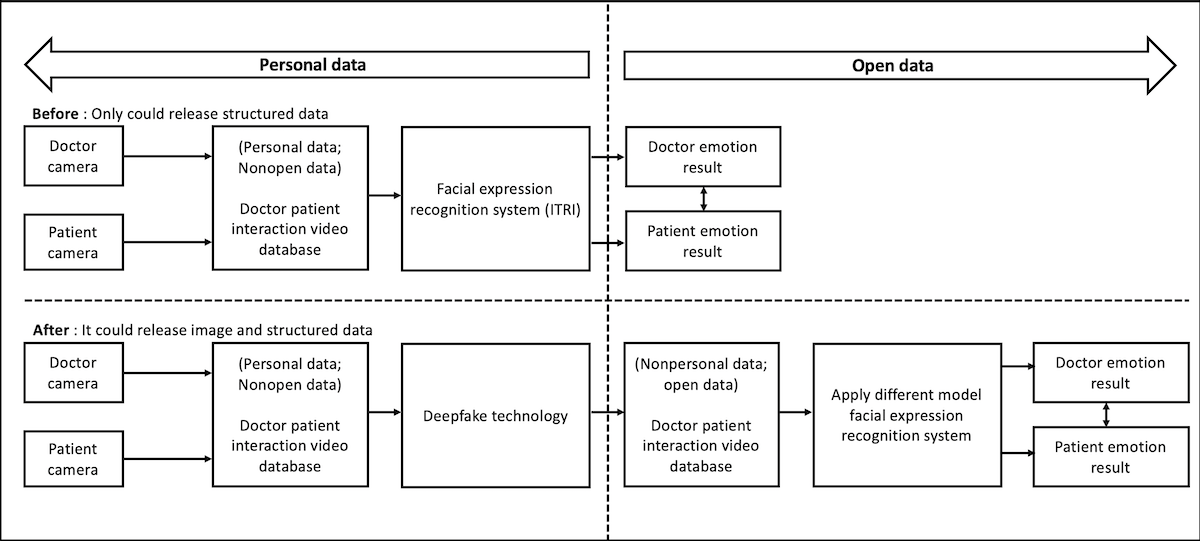
The facial emotion recognition system workflow. ITRI: Industrial Technology Research Institute.

Figure 1 illustrates the workflow of the FER system before and after proposing deepfake technology. First, we created synchronized recordings by using 2 cameras to capture doctor-patient interactions in the dermatology outpatient clinic. We assumed that the face was the most relevant and accessible channel for nonverbal communication in health care [6]. Therefore, we then used a facial expression recognition system developed by the Industrial Technology Research Institute to detect emotions and analyze the emotional changes of the doctors and patients across time. This facial expression recognition system has been deployed using training data from 28,710 Asian face images and has an accuracy of 95% for the extended Cohn-Kanade data set [35].

We identified facial expressions by using the main points of an individual’s face (eg, eyes, eyebrows, the tip of the nose, lip corners, etc) to track facial movement. This allowed us to observe the emotional experiences of the doctors and patients when they expressed the following seven facial expressions: anger, disgust, fear, neutral, happiness, sadness, and surprise. The system then provided a summary of the emotional changes of both the doctors and the patients with a temporal resolution of up to 1 second. Additionally, our model managed to filter out any irrelevant face targets (ie, faces other than those of the doctors and patients). Finally, the summary results of the doctor and patient emotion analyses were used as a reference data set to develop artificial empathy. The system then created recommendations, so that doctors could provide an immediate response based on patients’ situations.

It should be noted however that our artificial empathy reference data training set was built by using limited face recognition data sets. Therefore, we tried to improve the model by proposing the use of open data from a clinical encounter video manipulated by deepfake technology, which can enable medical data sharing without violating patient privacy. Furthermore, these open data allowed us to connect with real-world clinical encounter video data sets, so that we could use different model facial expression recognition systems to analyze patients’ and doctors’ emotional experiences (Figure 1).

### Ethics Approval

Our study was approved by Taipei Medical University (TMU)-Joint Institutional Review Board (TMU-JIRB No: N201810020).

## Results

The clinical encounter video—the source of our face recognition data set—consists of video data from 327 patients—208 female patients and 119 male patients (age: mean 51, SD 19.06 years). The average consultation time on the recorded video was 4.61 (SD 3.04) minutes; the longest duration of a consultation was 25.55 minutes, and the shortest was 0.33 minutes. Our artificial empathy algorithm was developed by using FER algorithms. This algorithm learned a range of patient emotions by analyzing expressions, so that doctors could provide an immediate response based on patients’ emotional experiences. In general, this FER system achieved a mean detection rate of >80% on real-world data.

**Figure 2:**
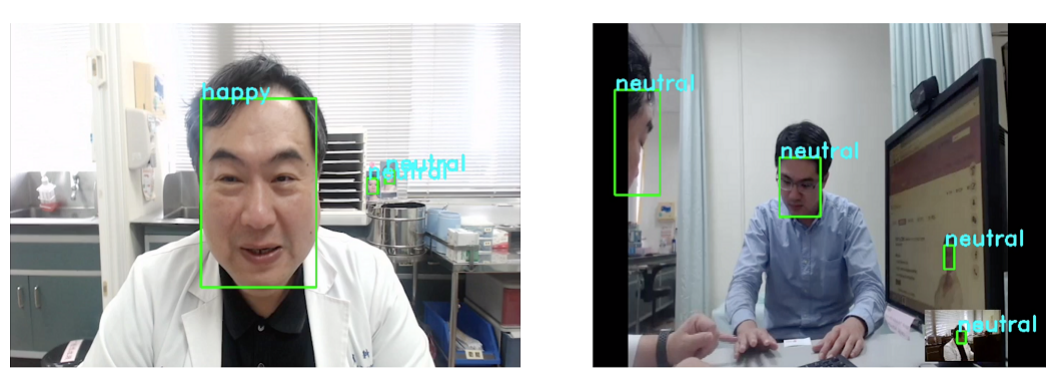
Screenshots of the recorded video simulation of the doctor-patient relationship in the dermatology outpatient clinic.

Our face recognition data set for artificial empathy was solely based on basic emotions. The system evaluation reported expressions of anger, happiness, disgust, and sadness, which were more likely to be expressed by the doctors than by the patients (*P*<.001). Moreover, patients also tended to more commonly express neutral emotions and surprise when compared to doctors (*P*<.001). The overall emotions of the doctors were dominated by emotions of sadness (expressions: 8580/17,397, 49.3%), happiness (expressions: 7541/17,397, 43.3%), anger (expressions: 629/17,397, 3.6%), surprise (expressions: 436/17,397, 2.5%), and disgust (expressions: 201/17,397, 1.2%), whereas the emotions of patients consisted of happiness (expressions: 5766/12,606, 45.7%), sadness (expressions: 5773/12,606, 45.8%), surprise (expressions: 890/12,606, 7.1%), and anger (expressions: 126/12,606, 0.9%). Figure 2 illustrates the emotional expressions of both doctors and patients. The system used the results of the emotion analysis to remind the doctors to change their behaviors according to patients’ situations, so that the patients felt like the doctors understood their emotions and situations.

**Figure 3:**
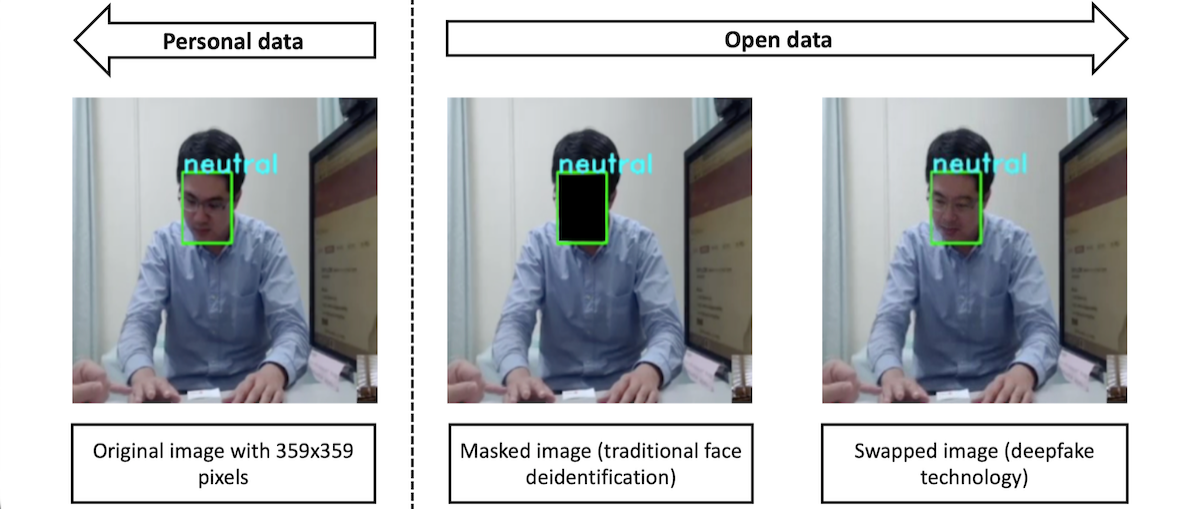
Comparison between traditional face deidentification and face swapping by using deepfake technology on an image of a patient's face.

The original face recognition data set consists of personal data (ie, patients’ faces). However, we can only release the results of the emotional expression analysis as a reference for the development of artificial empathy. As noted previously, our approach only involved using a small amount of training data (only Asian face images). Therefore, to improve model performance, we need to anonymize the clinical interaction video by performing face deidentification. Face deidentification allows us to share our face recognition data set as open data for clinical research. To enable face image data sharing, a researcher can perform traditional face deidentification techniques, such as masking an image by covering a patient’s face region with a colored box (Figure 3).

Of note however, as our research aims to develop artificial empathy to support good doctor-patient relationships, the masking method cannot be performed, as it is very difficult to validate masked images with the results of an emotion expression analysis. Deepfake technology offers a method for swapping a patient's original face with another from an open-source face data set to generate an unrecognizable image with similar expressions and attributes to those of the original face image. This face swapping method can be adopted for use with the face recognition reference data set for our artificial empathy algorithm to avoid violating patient privacy and ethical concerns. We adopted video deepfake technology based on face swapping (Figure 3), which was proposed in the first order motion model for image animation [36]. This approach involved adopting a novel deep learning framework for image animation known as *Monkey-Net* and modifying it by using a set of self-learned key points combined with local affine transformations [36]. This framework enables a dense motion transfer network to generate a video in which the source image is animated according to a given driving video sequence with complex motions [36]. Unlike the original GAN model, which relied on costly ground-truth pretrained models that resulted in the poor generation quality of image or video outputs, the first order motion model for image animation can handle high-resolution data sets with profile images and can thus become a reference benchmark model for our face recognition data set.

## Discussion

### Principal Findings

Our FER study revealed how doctors more commonly express emotions like anger, happiness, disgust, and sadness than patients. Because nonverbal messages like facial expressions contribute the most to the messages delivered in human interactions, doctors need to be more careful when expressing their emotions during clinical interactions. For example, doctors should never be used to expressing anger, disgust, or other negative emotions that represent poor communication skills, as this may ruin treatment goals and result in frustration for both patients and health care practitioners [6].

Positive emotions (eg, happiness) represent good communication skills, as they may help people understand how another person feels and what they think and allow people to understand each other better [37]. Furthermore, positive emotions can help build patients' trust in their doctors [38]. Trust from a patient’s perspective refers to the acceptance of a vulnerable situation in which patients believe that doctors will provide adequate and fair medical care to help them based on their needs [39]. When patients trust their doctors, they are more likely to share valid and reliable information related to their condition, acknowledge health problems more readily, comprehend medical information efficiently, and comply with treatment plans accordingly [39]. They also tend to seek preventive care earlier and return for follow-up care, which may prevent further disease complications [39].

In addition to physicians’ medical knowledge and clinical skills, patients’ perceptions of physicians’ ability to provide adequate information, actively listen, and empathize are believed to be associated with patient satisfaction and trust [3]. A physician's capability to exhibit effective communication skills and provide empathic care is beneficial for patients in terms of improving good doctor-patient relationships and for the physicians themselves, as these factors can increase job performance satisfaction and lower the risk of stress and physical burnout among physicians [40]. Empathic care may also reduce the rate of medical errors and help to avoid conflict with patients [38].

We believe that our FER system and face recognition data set can serve as a decision support system that can guide doctors when a patient requires special attention for achieving therapeutic goals. For example, if doctors express a negative facial expression (eg, anger, disgust, and sadness), the system will remind them to change their facial expressions. Moreover, if a patient also expresses a negative facial expression, the system will suggest that the doctor should use a different approach to accommodate the patient’s emotional situation. Based on our results, the major shortcoming that we need to address is that FER technology relies on the quality of data training and the quantity of training data [26,32]. We believe that in the future, we can improve the system’s precision and accuracy by collecting more data from more subjects with various sociodemographic backgrounds. This is only possible if we adopt deepfake technology (eg, GANs), which can learn the facial features of a human face on images and videos and replace it with another person's face [41]. Thus, deepfake technology can replace a patient’s face image and create fake face images with similar facial expressions in videos. With the use of deepfake technology, the recorded video database of outpatient doctor-patient interactions will become more accessible. Applying deepfakes to deidentify FER data sets may benefit the development of artificial empathy, as this approach may not violate the privacy and security of interpersonal situations.

Similar to our study, a recent study reported using deepfake technology to generate open-source, high-quality medical video data sets of Parkinson disease examination videos to deidentify subjects [32]. This study also applied the face swapping technique and real-time multi-person system to detect human motion key points based on open-source videos from the Deep Fake Detection data set [32]. Meanwhile, our approach involved using a self-supervised formulation consisting of self-learned key points combined with local affine transformations [36]. We believe that this self-learned model could preserve the represented emotional states of people in the original face recognition data set.

Our study has some limitations. First, our approach only involved using a single information modality—video deepfakes—which could have resulted in inaccurate emotion classification. In the future, we can combine both video and audio deepfakes to better represent the emotional states of a target person. Second, moral and ethical concerns need to be considered when using deepfake technology for the deidentification of medical data sets. However, our study highlighted the positive ways of using deepfakes for privacy protection when using face recognition data sets in medical settings. Thus, instead of raising an ethical problem, this study will help prevent the use of deepfakes for malicious purposes and encourage their use in medical applications.

### Conclusion

We propose using an open data set of clinical encounter videos as a reference data training set to develop artificial empathy based on an FER system, given that FER technologies rely on extensive data training. Yet, due to privacy concerns, it has always been difficult for researchers to acquire a face recognition data set. Therefore, we suggest the adoption of deepfakes. Deepfake technology can deidentify faces in images or videos and manipulate them so that the proper target face becomes unrecognizable, thereby preventing the violation of patient privacy. Such technology can also generate the same facial expressions as those in the original image or video. Therefore, this technology might promote medical video data sharing, improve the implementation of FER systems in clinical settings, and protect sensitive data. Furthermore, deepfake technology will further enhance the potential use of artificial empathy in helping doctors provide empathic care based on patients’ emotional experiences to achieve a good doctor-patient therapeutic relationship.

## Abbreviations

AI: artificial intelligence
FER: facial emotion recognition
GAN: generative adversarial network
